# Analysis of unmapped regions associated with long deletions in Korean whole genome sequences based on short read data

**DOI:** 10.5808/GI.2019.17.4.e40

**Published:** 2019-12-20

**Authors:** Yuna Lee, Kiejung Park, Insong Koh

**Affiliations:** 1 Department of Biomedical Informatics, Hanyang University, Seoul 04763, Korea; 2 Cheonan Industry-Academic Collaboration Foundation, Sangmyung University, Cheonan 31066, Korea; 3 KIOST School, University of Science and Technology, Daejeon 34113, Korea

**Keywords:** deletion, Korean, structural variation, unmapped region, whole genome sequencing

## Abstract

While studies aimed at detecting and analyzing indels or single nucleotide polymorphisms within human genomic sequences have been actively conducted, studies on detecting long insertions/deletions are not easy to orchestrate. For the last 10 years, the availability of long read data of human genomes from PacBio or Nanopore platforms has increased, which makes it easier to detect long insertions/deletions. However, because long read data have a critical disadvantage due to their relatively high cost, many next generation sequencing data are produced mainly by short read sequencing machines. Here, we constructed programs to detect so-called unmapped regions (UMRs, where no reads are mapped on the reference genome), scanned 40 Korean genomes to select UMR long deletion candidates, and compared the candidates with the long deletion break points within the genomes available from the 1000 Genomes Project (1KGP). An average of about 36,000 UMRs were found in the 40 Korean genomes tested, 284 UMRs were common across the 40 genomes, and a total of 37,943 UMRs were found. Compared with the 74,045 break points provided by the 1KGP, 30,698 UMRs overlapped. As the number of compared samples increased from 1 to 40, the number of UMRs that overlapped with the break points also increased. This eventually reached a peak of 80.9% of the total UMRs found in this study. As the total number of overlapped UMRs could probably grow to encompass 74,045 break points with the inclusion of more Korean genomes, this approach could be practically useful for studies on long deletions utilizing short read data.

## Introduction

Although there is no established definition of size for structural variations in the genome, they are generally accepted as being longer than 50 bp. They account for about 1% of all variants. Structural variations affect the diversity of phenotype expression and induce various diseases [1]. There are generally four ways to find structural variations from the read data produced by next generation sequencing (NGS) platforms [1,2].

The first method is to find structural variations using the read depth. When reads are mapped to a reference genome, the read depth can be estimated. CNVnator [3], which was applied in the 1000 Genomes Project (1KGP) for whole genome sequences (WGS), is a representative analytical tool for this method.

The second method uses paired-end reads, and it is based on the span and orientation of paired-end reads. If the distance between the mapped positions of two reads is longer than the insert size (i.e. the length of the unsequenced region), it may indicate an insertion. If the distance is shorter than the insert size, it may indicate a deletion. The analysis tools available for use with the paired-end read method include BreakDancer [4], TIGRA-SV [5], Delly [6], and so on. Each analytical tool differs in the way it performs filtering or statistical estimation.

The third method uses split-reads. When reads are mapped to a reference genome, a read may be split into two parts, breaking the alignment to the reference. A gap in the reference is indicative of a deletion, while a stretch in the read is indicative of an insertion. The analysis tools which function by using the split-read method extract a CIGAR string from the SAM/BAM file and find break points from different types of data. Some examples of the former are Breakpointer [7], Delly [6], LUMPY [8], TARDIS [9], and so on.

The last method is to detect structural variations based on split-contigs produced by *de novo* assembly [1,2]. Contigs are created by merging and ordering short reads to reassemble the original sequences in a *de novo* assembly. Some popular tools that utilize this technique are TIGRA-SV [5], BreaKmer [10], HYDRA [11], and NovelSeq [12].

With the advent of NGS technology, the cost of genome analysis has fallen sharply; this has led to the mass production of NGS data. NGS data have been mostly produced as short read data using the Illumina platform. Indel or single nucleotide polymorphism studies have been actively performed and analyzed. Detecting long deletions/insertions, however, is very difficult using short read data. Recently, long read data from the PacBio and Nanopore platforms have made it easier to find long deletions/insertions, but their disadvantage comes in the form of their significantly higher production costs.

In contrast to the previous method used to detect structural variations using long read data, in this study, we used short read data of Korean WGS produced using the Illumina platform, which are more publicly available. We want to construct a method to find unmapped regions (UMRs) that have a high possibility of including long deletions. After confirming the distribution of UMRs in each individual, we compared the UMRs of 40 normal Koreans and found useful data about the long deletions of the Korean WGS through the distribution of union of UMRs (UUMRs) and common UMRs (CUMRs; i.e. intersection of UMRs). Because our data set is limited in sample number, the aim of this study was to confirm the possibility of detecting long deletions through the lens of UMRs collected with short read data.

## Methods

In this study, 40 normal Korean WGS were used. The data set consisted of 20 WGS from the Korea National Institute of Health (KNIH) and 20 WGS from the Korean Personal Genome Project (KPGP), and the study was approved by the Institutional Review Board (IRB). The data generated by KPGP are open to the FTP sites of the Genomics Institute (TGI) and the Korean Bioinformation Center (KOBIC) (ftp://ftp.kobic.re.kr//pub/KPGP/2017_release_candidate/WGS). The WGS data set was composed of paired end reads generated by the HiSeq Illumina platform (Illumina, San Diego, CA, USA): the 20 samples of genomic data from KNIH (read length = 101 bp, the average mapping depth = 30×) and the 20 samples of genomic data from KPGP (read length = 150 bp, the average mapping depth = 45×).

We used Human Build 37 (GRCh37) of the Genome Reference Consortium as the reference genome. The data are open to the FTP sites of the European Bioinformatics Institute (EBI) (ftp://ftp.1000genomes.ebi.ac.uk/vol1/ftp/pilot_data/data) and the National Institute of Health (NIH) (ftp://ftp.ncbi.nlm.nih.gov). Forty normal Korean genome sequences obtained using the NGS method were used for analysis after data preprocessing. First, we confirmed the read quality of raw data. During the amplification of DNA fragments on the Illumina platform, some reads may duplicate at the same position or low-quality reads may be produced. Therefore, the quality of each read was checked using FastQC (v0.11.2) [13] before analysis. Sickle (v.1.33) [14] was used to perform a trimming process to remove the attached adapters from the amplified DNA fragments.

The reference genome (build ver GRCh37) [15] must be indexed for analysis. The indexing process was performed using BWA -a bwtsw (v.0.7.12) [16,17]. The 40 data samples and indexed reference genome data were prepared for the mapping process by aligning them using the BWA-MEM (v.0.7.12) [16,17]. After the mapped file was sorted and indexed, duplicate reads were removed by using MarkDuplicates, one of the options provided by Picard (v.1.96) [18]. The 20 data samples from KPGP used in our analysis were divided and uploaded into FTP sites. In order to speed up the work, after performing the above processes, SAMtools (v.1.3.1) [19] was used to merge the split files into one file.

The process performed to find the UMRs was as follows. First, in order to find the UMRs for each chromosome, it was necessary to divide the BAM files by chromosome. The newly created files were indexed before moving on to subsequent operations. SAMtools (v.1.3.1) [19] was used to change header information, index files, and divide files by their respective chromosome number.

**Fig. 1.**
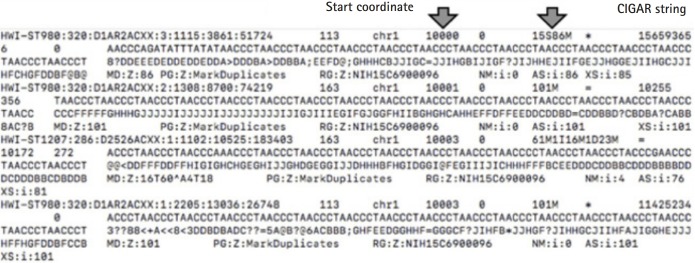
Start coordinates and CIGAR strings: mapping starting point and mapping information. We divided the BAM files by chromosome number.

**Fig. 2.**
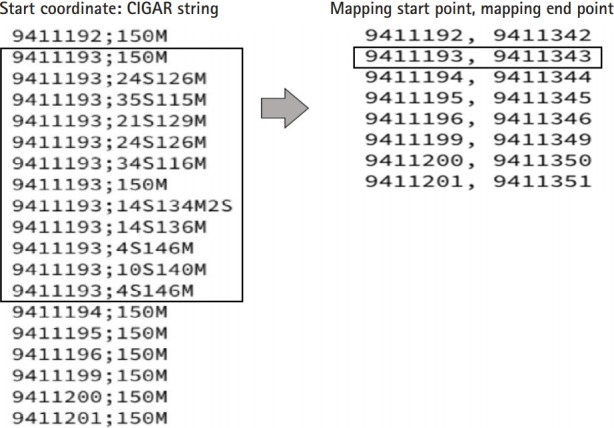
Extracting the mapping start point and the mapping end point: If the mapping start point is the same, the mapping end point is predicted by adding a large number of M values.

**Fig. 3.**
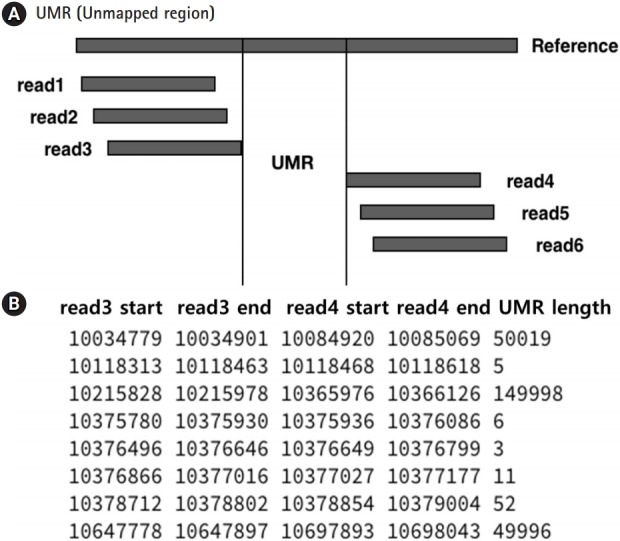
Principle of unmapped region (UMR) detection. (A) Read1 and read2 overlap when mapped. This is an object that is not considered for UMR. However, in the case of read3 and read4, since the mapping position does not overlap, the distance between the end point of read3 and the start point of read4 can be called the UMR. (B) Only read3 and read4 are collected, and the UMR length is estimated in the actual data.

In the BAM files for each chromosome, information in the 4th and 6th columns indicating the start coordinates and the CIGAR string [19] could be extracted (Fig. 1). Using the two kinds of extracted information, the points where the mapping of each read starts and ends were found (Fig. 2). The list was sorted only by start values. If the interval of two consecutive mapping end points is longer than the read length reflecting the mapping information, the two reads may not overlap. After collecting these non-overlapping reads, we found the intervals. In this study, these are indicated as the UMRs (Fig. 3). Actually, multiple mapping for a read was not treated.

Because there are genes that are essential but could be difficult to map due to many repetitions, the human genetic information provided by the National Center for Biotechnology Information (NCBI) was used to remove the gene locations in the UMRs. All the UMRs that overlapped with gene locations were removed. For example, the position of the human leukocyte antigen-A (HLA-A) gene is 6: 29,942,469–29,945,883 (from NCBI). If the position of a UMR such as 6: 29,942,460–29,945,875, overlapped with the HLA-A gene position, we removed this UMR. Actually, we calculated both data with and without gene locations.

**Fig. 4.**
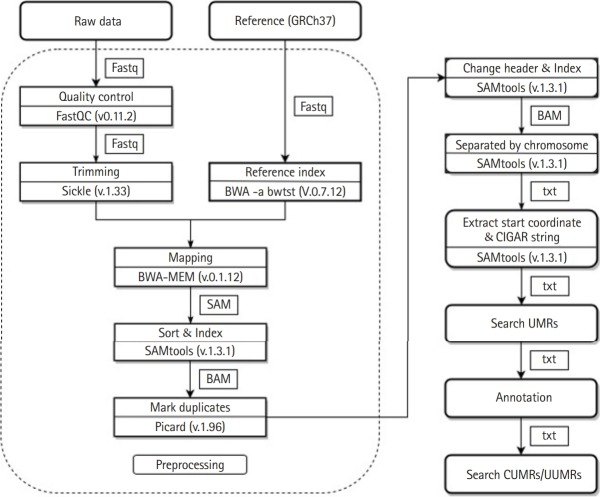
The Pipeline to find unmapped regions (UMRs) from Korean whole genome sequences: After the quality of each read was checked using FastQC (v0.11.2) [13], adapter trimming was performed using Sickle (v.1.33) [14]. A reference genome was indexed using BWA -a bwtsw (v.0.7.12) [16,17]. After mapping using BWA-MEM (v.0.7.12) [16,17], the sorting and indexing processes were performed using SAMtools (v.1.3.1) [19] in the order of the start coordinates. MarkDuplicates of Picard (v.1.96) [18] was used to remove redundant reads. The mapping starting points and CIGAR strings were extracted and the UMRs were found. Annotation was performed by removing all the UMRs overlapped with the gene locations. We searched union of UMRs (UUMRs), which are unions of UMRs, and common UMRs (CUMRs), which are intersections of UMRs.

All UMRs found in the 40 Korean samples were called UUMRs. We performed the filtering process on the UMRs of the 40 samples and obtained the UUMRs of the 40 Korean samples. Using this information, we were able to compare our data with the break points provided by the 1KGP to find candidate regions that could actually be deletions. Contrasting the deletions with insertions [20] determines the degree to which UMRs are associated with deletions. If there is structural variation in any section, the deletion and insertion may occur simultaneously [20]. This is because all of the break points of the 1KGP were used for analysis. Because the break points of the 1KGP contain all of the break points from the complete data set [20], the UMRs found in the data for the 40 Koreans based on the break points of the 1KGP were compared 1:1 to find UUMRs. After confirming that the UMRs were not accidental results, we determined the CUMRs in the Korean genomes. By comparing all of the starting and ending points from the Korean UMRs, we found consistently UMRs in all genome data (Fig. 4).

### Ethics

This study was approved by the Institutional Review Board (IRB) of Hanyang University (HYI-17-240-1, A bioinformatics study for detecting disease genetic markers based on NGS genome data analysis) with a waiver of informed consent.

## Results

### Selecting unmapped reads from samples

The average number of UMRs found within the 40 genomes was 36,042. An average of 47,793 UMRs were found in the 20 WGSs from KNIH compared to 24,291 UMRs found in the 20 WGSs from KPGP. The discrepancy between the two data sets was presumed to reflect the differences in the read counts and mapping depth. We estimated the read lengths and the mapping depths of the KNIH and KPGP data. The average read lengths from the KNIH and KPGP data were 101 bp and 150 bp, respectively, and the average mapping depths of coverage were 30 and 45, respectively. After mapping, we analyzed the frequency and distribution of UMRs for intergenic regions as well as for whole genome. The average numbers of filtered UMRs in the KNIH data (with short-read length and low mapping depth) and KPGP data were similar (3,119 in KNIH vs. 2,825 in KPGP) for intergenic regions.

### Union of unmapped regions

**Fig. 5.**
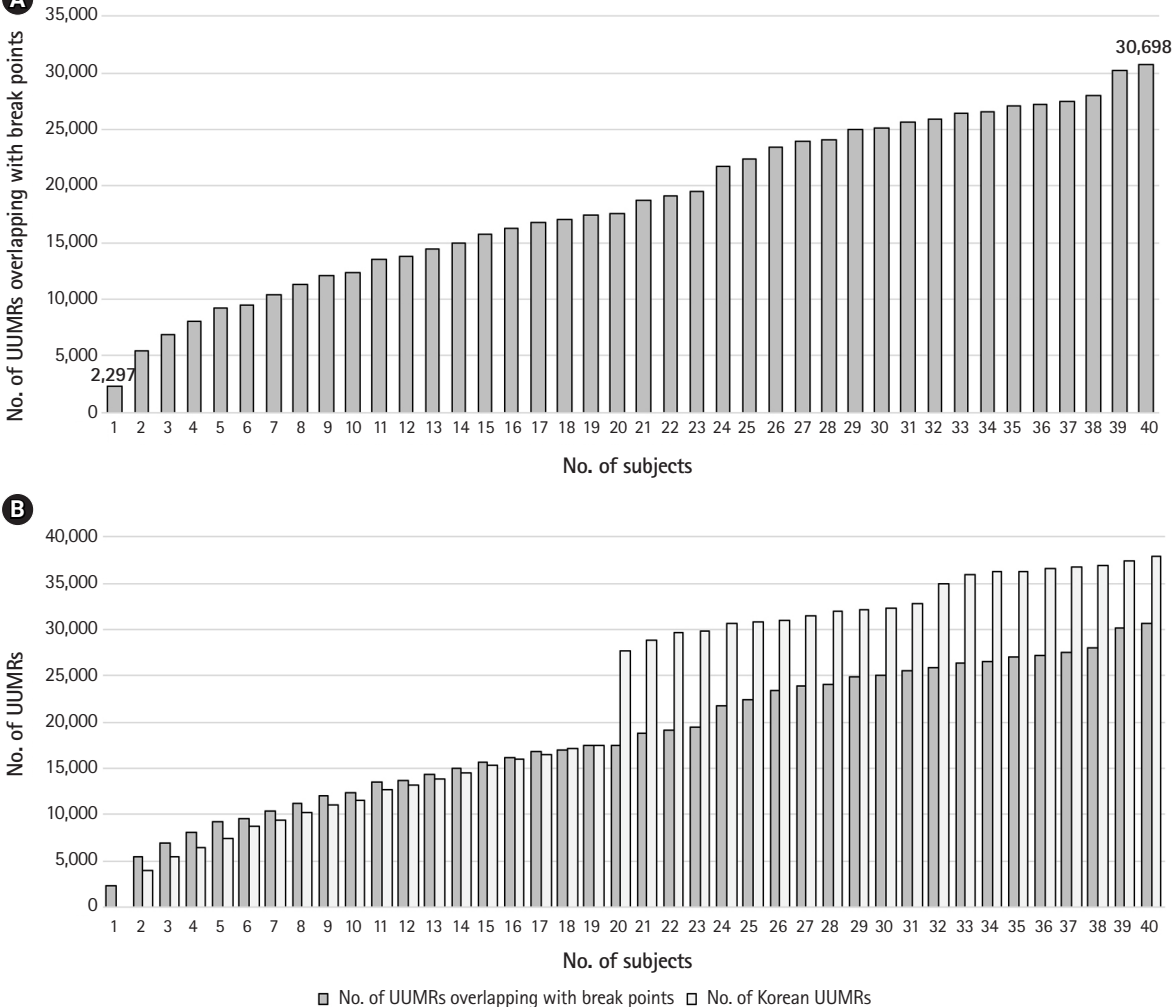
Distribution of union of unmapped regions (UUMRs) by the number of compared subjects. (A) An increasing number of the UUMRs of Korean genomes overlapped with the break points of the 1000 Genomes Project as the number of compared subjects increased. This increase is relatively gradual. (B) UUMR distribution by the number of 40 Korean subjects superimposed with (A): Since the two distributions show similarly increasing trends (although showing two different Korean genome resources), the UUMRs are believed to closely reflect deletions.

The data distribution for the 40 Korean UUMRs shows that an increase in the number of UUMRs is related to an increase in the number of samples (Table 1). Considering the break points provided by the 1KGP [20], the UUMRs created by excluding the gene locations should be mapped in the UMRs of 40 Koreans. The processed results are shown in Fig. 5. The number of UUMRs increases as the number of subjects compared with the 1KGP increases from 1 to 40. For example, in the case of one sample, 2,297 UUMRs overlapped and in the case of 40 samples, 30,698 UUMRs overlapped with the break points of the 1KGP data.

Although the accuracy of the analysis using individual UMRs is less precise, as the number of compared subjects increases, the number of UUMRs may approximate the number of break points (i.e. 74,045) provided by the 1KGP. Therefore, it may be predicted that the UUMRs can be interpreted as real deletions. Analyzing 1,000 or more Korean genomes would clarify if this approach is viable for searching for long deletions. This would be a highly relevant finding as we are attempting to understand deletions within Korean genomic data.

### Common unmapped regions

We define CUMR as the UMRs overlapping with one or more bases. In order to overcome the possible limitation of the deletion detection method utilizing UMRs, we also observed CUMRs in the 40-genome data set (Table 2). Although the genomes are from two different resources (KNIH and KPGP), the results show a similar ratio. The number of CUMRs within all 40 Korean WGS data was 284. In the data from KNIH, the number of CUMRs across all 20 samples was 1,577 and the number of CUMRs in the data produced by KPGP was 440. The average ratio of CUMRs to UMRs in the KNIH data was about 3.3% (1,577/47,793), whereas it was 1.8% (440/24,291) in the KPGP data. When we randomly selected 10 WGS data from each of two data sets, the ratio was about 1.4% (Table 2). It is predicted that the average number of CUMRs in Korean genomes probably decreases as the number of subjects increases over 40, but the ratios probably stay similar in all Korean genome data sets.

## Discussion

$D=\frac{R\times L}{G}=\mathrm{coverage}\;\mathrm{depth}\;\alpha =\frac{D}{G}\;=\;\mathrm{Probability}\;\mathrm{of}\;\mathrm{starting}\;\mathrm{mapping}\;\mathrm{on}\;\mathrm{the}\;\mathrm{reference}\;\mathrm{genome}\;\mathrm{by}\;\mathrm{chance}\;P=\frac{\alpha {\left(1-\alpha \right)}^{L\;+\;\mathrm{average}\;\mathrm{UMR}\;\mathrm{length}-1}}{L\;+\;\mathrm{average}\;\mathrm{UMR}\;\mathrm{length}}\times G\;=\;\mathrm{Probability}\;\mathrm{that}\;\mathrm{UMRs}\;\mathrm{occur}\;\mathrm{when}\;a\;\mathrm{read}\;\mathrm{if}\;\mathrm{mapped}\;\mathrm{randomls}$

, where, R is the number of reads, L is read length, and G is the length of reference genome.

If we apply the abovementioned equation to map the reads onto the reference genome, in case of sample NIH15C6900096, which shows a higher number of UMRs (65,658) than the other 39 Korean samples, the probability (p) that UMRs occur when a read is mapped randomly is 0.0272. Since the mapping depth of the sample is 30×, the probability of being randomly mapped with all the reads is 1.089 × 10^-47^(=0.0272^30^). It indicates that the reads were mapped in place and that the UMRs were not found by chance even in case of the sample with the highest UMR counts (NIH15C6900096). Although they are not accidentally discovered UMRs, it cannot be said that the UMRs found in this study are all long deletions. Even if we did not consider all the variables that could occur, it is significant that for a certain ratio, the UMRs of Korean genomes coincided with the break points of the deletions found in the 1KGP (Fig. 5). Thus, we believe that the proposed method can estimate actual deletions.

We may assume that the unfiltered UMRs explain long deletions after the processing step of reducing unmapped reads by loosening the mapping parameters. The filtering process described earlier, by which gene region UMRs are removed, supports the validity of this assumption. We predict that in mapping under the default conditions of BWA-MEM, the UMRs in the positions of some genes (which are difficult to be mapped) can be removed by alleviating the mapping parameters. It is still necessary to increase the size of the data set to get more accurate results in order to overcome weakness of using short read data sets.

In this study, we focused on detecting deletions using UMRs, so we searched for overlapping regions between the UUMRs of the Korean population and the break points (i.e. known deletions) of the 1KGP and predicted the overlapped regions as possible deletions. However, the predicted parts are located only on the reference genome. If the Korean and 1KGP genomes had been separated from the same ancestor, the predicted parts can be described as insertions within the reference genome. We expect that further study involving the comparison of the Korean genome data, 1KGP data, and the reference genome may provide essential clues for detecting insertions.

Further analysis of large scale Korean short read genome data, including KPGP data, may increase the reliability of this detection method. It is also meaningful as a method to find characteristics in racial distribution of deletions, if the racial information on the break points can be secured. It is expected that the detection method proposed in this study might help researchers implement initial ideas for studying structural variations, including long deletions. More precise manipulation of reads, including cleaning low quality region before mapping, could be necessary for improvement.

The UMR detection method described in this study is not a new one, but the analysis approach for detecting structural variations is different from the ones described in previous studies, in that this study used short read data rather than long read data. In other words, the analysis approach used in this study is more meaningful than the method itself. Although this study is simple, limited by data size, and only applied to the Korean genome, we expect that applying the method described by us to the genomes of other ethnic populations will produce similar results for detecting long deletions and, thus, show the validity of this approach.

## Figures and Tables

**Table 1.** Distribution of UUMRs in 40 Korean subjects

No. of subjects	No. of Korean UUMRs	No. of subjects	No. of Korean UUMRs			
2	4,036	22	29,696			
3	5,387	23	29,898			
4	6,397	24	30,630			
5	7,367	25	30,793			
6	8,766	26	30,952			
7	9,481	27	31,544			
8	10,319	28	32,027			
9	10,989	29	32,197			
10	11,488	30	32,314			
11	12,770	31	32,749			
12	13,178	32	35,035			
13	13,886	33	36,034			
14	14,504	34	36,213			
15	15,402	35	36,342			
16	15,976	36	36,691			
17	16,561	37	36,804			
18	17,174	38	36,930			
19	17,547	39	37,366			
20	27,699	40	37,943			
21	28,882					

UUMR, union of unmapped regions.

**Table 2.** Distribution and ratio of Korean CUMRs by the numbers of people and samples

	All samples	KNIH	KPGP	Random1	Random2	Random3	Random4	Random5
		20	20	KNIH 10	KNIH 10	KNIH 10	KNIH 10	KNIH 10
		samples	samples	KPGP 10	KPGP 10	KPGP 10	KPGP 10	KPGP 10
CUMRs	284	1577	440	490	521	511	496	473
CUMRs/UMRs (%)	0.8	3.3	1.8	1.3	1.5	1.4	1.4	1.3

CUMR, common unmapped region; KNIH, Korea National Institute of Health; KPGP, Korean Personal Genome Project; UMR, unmapped region.
